# Shapes of the uterine cavity are different in women with polycystic ovary syndrome

**DOI:** 10.1002/rmb2.12508

**Published:** 2023-02-23

**Authors:** Shunsaku Fujii, Takaaki Oguchi

**Affiliations:** ^1^ ef.clinic Aomori Japan

**Keywords:** androgens, infertility, polycystic ovary syndrome, ultrasound, uterine anomalies

## Abstract

**Purpose:**

A cross‐sectional study was conducted to evaluate differences in uterine morphology between women with or without polycystic ovary syndrome.

**Methods:**

The authors recruited 333 infertile reproductive‐age women including 93 with polycystic ovary syndrome diagnosed using the criteria of the Japanese Society of Obstetrics Gynecology‐2007. Shapes of uterine cavity were measured by transvaginal three‐dimensional ultrasound.

**Results:**

The polycystic ovary syndrome group had a significantly deeper indentation (2.2 ± 0.4 mm vs. 0.0 ± 0.2 mm, *p* < 0.0001) and a significantly more acute indentation angle (162.9 ± 2.2 deg vs. 175.2 ± 1.3 deg, *p* < 0.0001) than the control group.

**Conclusion:**

The depth and the apical angle of fundal indentation of uterine cavity are different in women with polycystic ovary syndrome.

## Graphical abstract

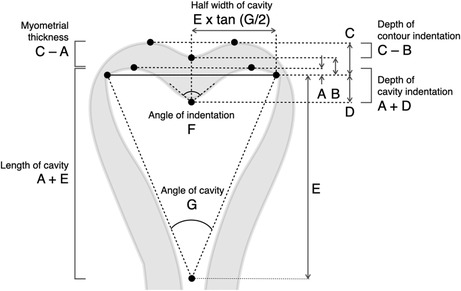

## 1 INTRODUCTION

Polycystic ovary syndrome (PCOS) is the most common—and increasing—cause of female infertility caused by irregular ovulation.^1^ The main features of PCOS in reproductive‐age women are oligo‐anovulation, polycystic ovarian morphology (PCOM), and hyperandrogenism. The current hypothesis for the pathogenesis of PCOS assumes prenatal androgen exposure in female fetuses, induced by high maternal levels of anti‐Müllerian hormone (AMH), which inhibit placental aromatase activity.^2^ There is increasing evidence that PCOS is of potential fetal origin and can be transmitted across generations.^3^, ^4^ As a sensitive biomarker for prenatal androgen exposure, the anogenital distance (AGD), the distance measured from the anus to the genital tubercle, has been recognized, and a longer AGD is related to PCOS,^5^ even in fetuses in utero.^6^ Other congenital features in women with PCOS are uterine anomalies arising from a defect in development of the Müllerian ducts. Although a high incidence of uterine anomalies in infertile patients with PCOS has been reported,^7^, ^8^, ^9^, ^10^, ^11^ the diagnostic criteria or classifications for uterine anomalies have not been unified, so the different criteria have resulted in different reported prevalence rates and types of anomalies. Therefore, we conducted this study to evaluate any differences in uterine morphology between women with or without PCOS by measuring uterine shapes in detail using three‐dimensional ultrasound (3D‐US).

## 2 MATERIALS AND METHODS

### 2.1 Study population

This cross‐sectional observational study was carried out in a private infertility clinic. Infertile women aged <40 years who visited our clinic from April 2021 to March 2022 were enrolled. All data used in this study were obtained during routine infertility examinations without any additional interventions. Venous blood samples for assaying the basal serum concentrations of luteinizing hormone (LH), follicle‐stimulating hormone (FSH), estradiol, and testosterone were collected during the first 5 days of spontaneous menstrual cycles or if the United States did not show large follicles (diameter > 9 mm) or a thick endometrium (>6 mm). Testing of blood samples was carried out in our laboratory using an Access 2 immunoassay system (Beckman‐Coulter). The diagnosis of PCOS was based on the criteria of the Japanese Society of Obstetrics and Gynecology (JSOG)‐2007.^12^ Briefly, patients who had both oligo‐anovulation and polycystic ovarian morphology (PCOM), and either hyperandrogenemia (total testosterone level > 0.47 ng/dL) or elevated serum LH (>7 mIU/mL) with normal serum FSH (3–8 mIU/mL), were diagnosed as PCOS. Patients with an elevated FSH level (>14 mIU/ml), or who had US‐visible tumorous lesions in a small pelvic cavity (e.g., fibroids, endometrial polyps, ovarian cysts, ovarian endometrioma, or adenomyosis), or had a history of uterine surgery (e.g., myomectomy or cesarean section), were excluded.

Transvaginal ultrasound was performed using an 3D‐US system (Voluson SWIFT; GE Healthcare Ultrasound) with volumetric transvaginal probes (RIC5‐9A‐RS) at the time when endometrial thickness was greater than 6 mm regardless of the day of the cycle. The 3D‐US datasets of each uterus were anonymized, except for each patient's identification number (ID), and stored for later measurements.

### 2.2 Measurements of the uterine cavity

**FIGURE 1:**
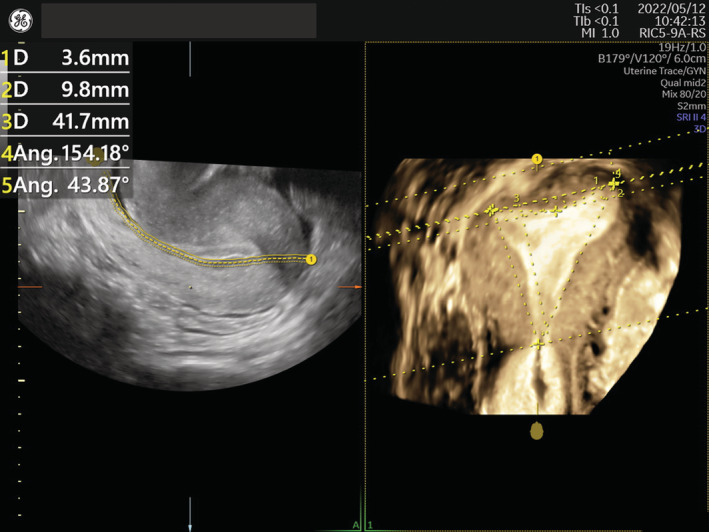
Example image obtained by 3D ultrasound system with volumetric transvaginal probes. Left: the mid‐endometrial line traced on a sagittal plane. Right: measures referring the line connecting the tubal ostium (indicated as yellow crosses) of each side of the uterus on a generated coronal plane.

**FIGURE 2:**
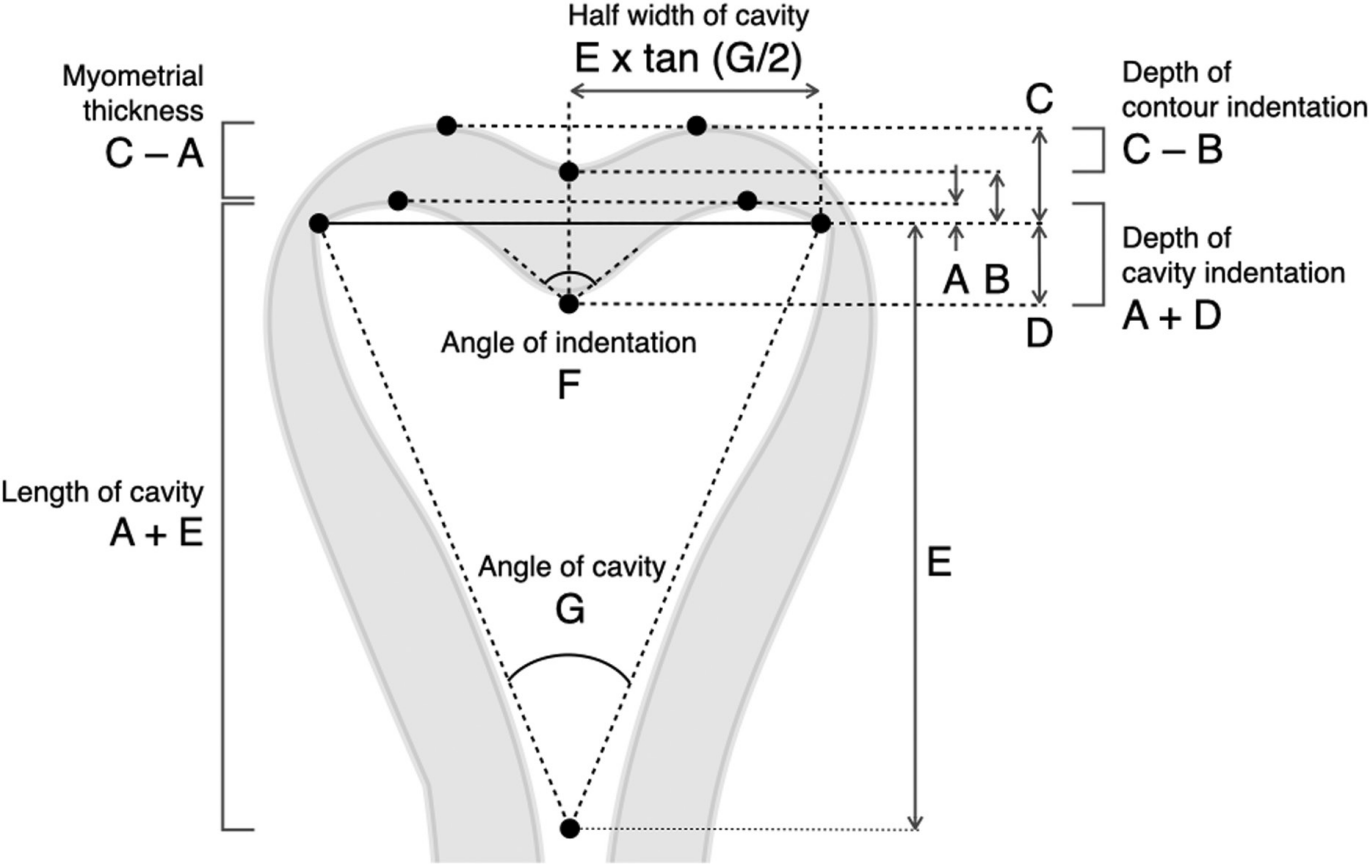
Measurements of uterine shape. The distance (in mm) was measured between the tubal ostial line (solid) and each of the following parallel lines: the line at the highest point of endometrial cavity (A), the lowest point of the fundal cleft of the uterus (B), the line at the highest point of the uterine contour (C), the line at the apex of indentation (D), and the line at the internal cervical os (E). The angle (in degrees) of indentation (F) and of cavity (G) were measured. The depth of indentation as (A + D), the myometrial thickness as (C − A), the indentation depth of the fundal cleft as (C − B), the length of cavity as (A + E), and the half‐width of cavity as [E × tan (G/2)] were calculated.

To minimize bias in measurement, measurements of uterine shapes were done collectively on stored anonymous 3D‐US datasets. We measured the uterine cavity shape on an accurate coronal plane, which was obtained by tracing the exact mid‐endometrial line on the touch‐panel screen of the 3D‐US system (Figure 1). The tubal ostial line (that connecting the tubal ostium of each side of the uterus) was used as a reference baseline (Figure 2). Using the “Distance 2 Line” mode of the 3D‐US system, used to measure the distance between two parallel lines, the distance between the tubal ostial line and each of the following parallel lines were measured in mm in the following order: the line at the highest point of the endometrial cavity (A); the lowest point of the fundal cleft of the uterus (B); the line at the highest point of the uterine contour (C), the line at the apex of indentation (D); and the line at the internal cervical os (E). Using the “Angle 3 Points” mode of the system, which is used to measure the angle between three points, the apical angle (in degrees) of indentation (F) was measured with both points close to each side of the indentation and its apex, and the angle of cavity (G) was measured pointing both tubal ostia and the internal cervical ostia. The depth of indentation was calculated as (A + D). The myometrial thickness was calculated as (C − A). The indentation depth of the fundal cleft was calculated as (C − B). The length of cavity was calculated as (A + E). The half‐width of cavity was calculated as [E × tan (G/2)]. Septate uterus was diagnosed based on the American Society for Reproductive Medicine (ASRM) classification‐2021,^13^ that is, septum length (equal to the length of cavity indentation; A + D) > 1 cm, septum angle (equal to the angle of indentation; F) < 90°, and depth of the fundal cleft <1 cm. The physical status and hormone levels of each patient were recovered from the medical records by checking their hospital ID.

### 2.3 Statistical analysis

We designed this study to have an alpha value of 0.05 and a power of 80% to detect 1 mm difference in values of ultrasound measurements between the groups. We calculated that at least a total of 372 subjects would need to be recorded, assuming that standard deviation of the length of uterine cavity in each group would be 3 mm, and 30% would be excluded by exclusion criteria. To compare the means across groups, Student's *t*‐test and analysis of variance (ANOVA) were used. Statistical analyses were carried out using JMP software (v. 15.2.1; SAS Institute Inc.). Data are represented as mean ± standard error (SE) for continuous variables. All tests were two‐tailed, and *p* < 0.05 was considered statistically significant.

## 3 RESULTS

Among 446 patients diagnosed with infertility who visited our clinic, 333 women who met the inclusion criteria were enrolled. Ninety‐three women were diagnosed with PCOS (the PCOS group), and other 240 women were not (the non‐PCOS group). In the PCOS group, means of basal LH levels and testosterone levels were significantly higher and means of FSH levels were significantly lower. Means of age, body mass index (BMI), and serum levels of estradiol were not different between the two groups (Table 1).

**TABLE 1:** Characteristics of the polycystic ovary syndrome (PCOS) and non‐PCOS groups.

	PCOS (n = 93)		Non‐PCOS (n = 240)		*p*
	M ± SE	[95% CI]	M ± SE	[95% CI]	
Age (years)	32.8 ± 0.5	[31.9–33.7]	33.8 ± 0.3	[33.1–34.4]	0.1316
BMI (kg/m^2^)	22.3 ± 0.4	[21.5–23.2]	21.5 ± 0.3	[20.9–22.1]	0.0968
LH (mIU/mL)	9.5 ± 0.5	[8.4–10.5]	5.4 ± 0.5	[4.5–6.3]	<0.0001
FSH (mIU/mL)	7.2 ± 0.2	[6.7–7.6]	7.9 ± 0.2	[7.5–8.3]	0.0147
Testosterone (ng/mL)	0.53 ± 0.02	[0.49–0.58]	0.33 ± 0.02	[0.29–0.37]	<0.0001
Estradiol (pg/mL)	62.1 ± 4.1	[53.9–70.3]	62.1 ± 3.5	[55.1–69.1]	0.9982

*Note*: Variables were compared using Student's *t*‐test and analysis of variance (ANOVA).

Abbreviations: BMI, body mass index; CI, confidence interval; FSH, follicle‐stimulating hormone; LH, luteinizing hormone; M, mean; SE, standard error of the mean.

**FIGURE 3:**
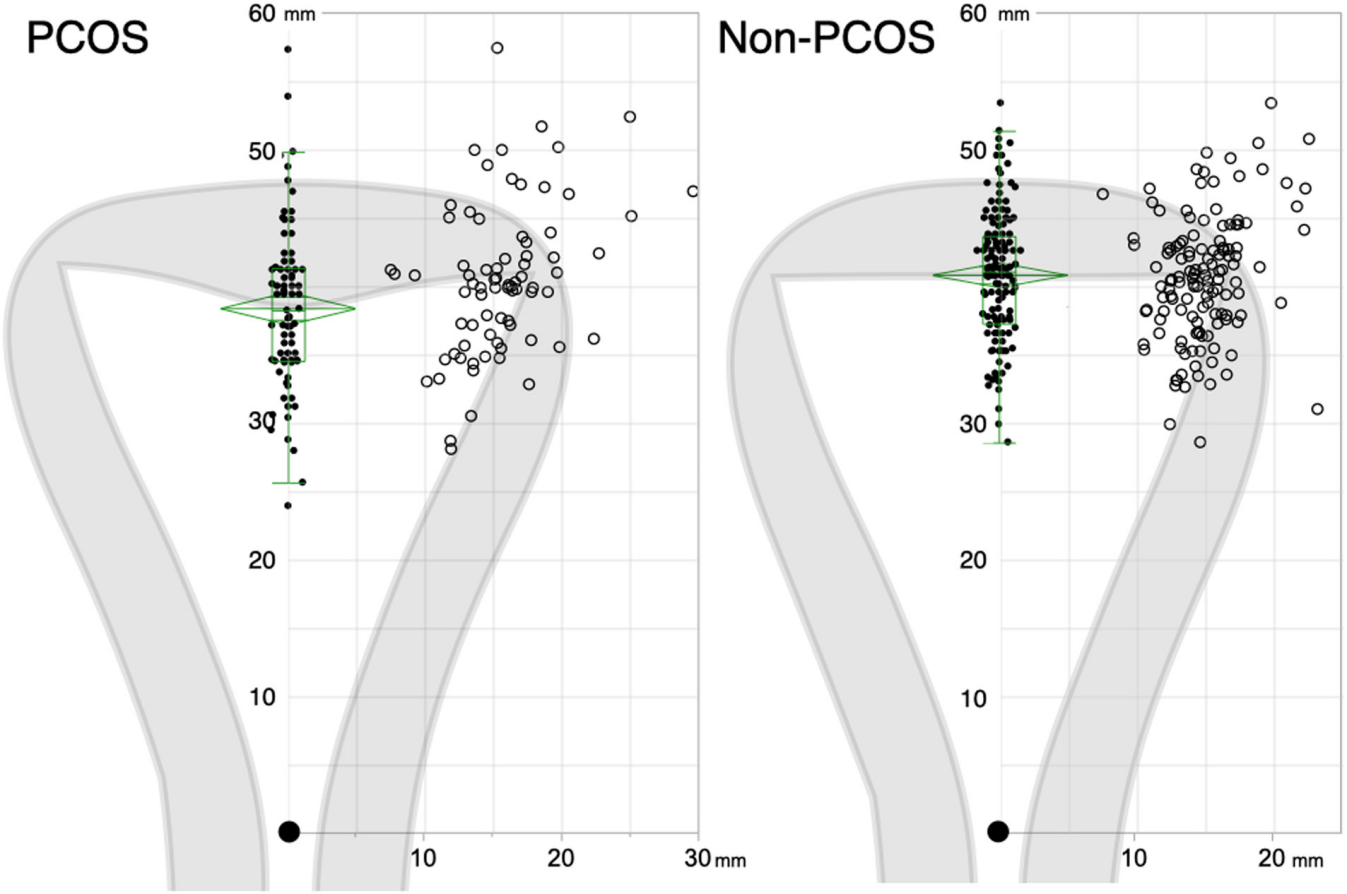
Average of uterine cavity shape. The average uterine cavity shapes of the PCOS and non‐PCOS groups, drawn according to the mean position of the tubal ostia and the apex of cavity indentation by setting the position of internal cervical os (closed large circles) as the origin of the coordinate axes. Open circles represent the positions of tubal ostia, and closed circles represent the apex of cavity indentation. The green diamond plots based on analysis of variance (ANOVA) denote the mean (the center line) and the 95% confidence intervals (CI) for each mean (the top and the bottom points of diamonds). The green boxes represent the distribution, and the center line denotes the median value (50th percentile), while the box contains the 25th to 75th percentiles of dataset. The whiskers mark the 5th and 95th percentiles. Left: PCOS group. Right: non‐PCOS group.

The length of cavity and half‐width of cavity were not different between the groups. Myometrial thickness was significantly thinner in the PCOS group. The fundal cleft was observed in only one patient with PCOS; therefore, we excluded the indentation depth of the fundal cleft from the analysis. The PCOS group had a significantly deeper indentation and a significantly more acute angle of indentation as compared with the non‐PCOS group. Septate uteri were found in three patients only in the PCOS group (Table 2). The average shape of the uterine cavity of the PCOS group generated by the mean position of the tubal ostia and the mean depth of cavity indentation resembled an arcuate uterus (Figure 3).

**TABLE 2:** Uterine shapes of the polycystic ovary syndrome (PCOS) and non‐PCOS groups.

	PCOS (*n* = 93)		Non‐PCOS (*n* = 240)		*p*
	M ± SE	[95% CI]	M ± SE	[95% CI]	
Length of cavity (mm) = A + E	41.0 ± 0.5	[39.9–42.1]	40.6 ± 0.3	[39.9–41.3]	0.5410
Half‐width of cavity (mm) = E × tan (G/2)	15.7 ± 0.3	[15.1–16.3]	15.1 ± 0.2	[14.8–15.5]	0.1019
Myometrial thickness (mm) = C − A	10.8 ± 0.2	[10.3–11.2]	11.4 ± 0.2	[11.1–11.7]	0.0322
Depth of cavity indentation (mm) = A + D	2.2 ± 0.4	[1.5–2.9]	0.0 ± 0.2	[−0.4–0.5]	<0.0001
Angle of indentation (deg) = F	162.9 ± 2.2	[158.6–167.3]	175.2 ± 1.3	[172.6–177.9]	<0.0001
Angle of cavity (deg) = G	42.0 ± 0.8	[40.5–43.5]	41.1 ± 0.5	[40.1–42.0]	0.2804
Septate uterus	3	(3.2%)	0	–	

*Note*: Variables were compared using Student's t‐test and analysis of variance (ANOVA).

Abbreviations: CI, confidence interval; M, mean; SE, standard error of the mean.

## 4 DISCUSSION

The etiology of PCOS is still not well understood because of its heterogeneity and complexity. Although genome‐wide association studies (GWAS) have found 21 genetic loci associated with PCOS,^14^, ^15^, ^16^ the genetic susceptibility only accounts for less than 10% of its heritability.^17^ Tata et al.^2^ demonstrated that high AMH levels in pregnant women with PCOS were implicated in the postnatal manifestation of PCOS in offspring. They confirmed that pregnant mice injected with AMH increased testosterone production and stimulated GnRH neurons leading to increased LH pulsatility, which were often found in normal‐weight PCOS subjects. They concluded that increase in both testosterone and LH passing across the placenta could be responsible for the epigenetic alteration in development of the fetal ovary. Alternatively, Risal et al.^3^ demonstrated that daughters of mothers with PCOS had five times risk to develop PCOS and prenatal androgen exposure made F_1_–F_3_ offspring developing PCOS‐like phenotypes with altering gene expression. Considering these findings, it is possible that DOHaD (developmental origins of health and disease) underlies the postnatal development of PCOS through an altered milieu in utero.

The commonly used diagnostic criteria for PCOS are 2003 Rotterdam criteria.^18^ Referring to the phenotype classification recommended by the NIH consensus panel 2012,^19^ subjects with hyperandrogenemia in this study correspond to phenotype A (hyperandrogenism + ovulatory dysfunction + PCOM), and those without hyperandrogenemia correspond to phenotype D (ovulatory dysfunction + PCOM). As uterine shapes were not different between phenotypes with or without hyperandrogenemia and were not correlated with any of hormone levels (not mentioned in this paper), it was difficult to suppose which factors contributed most to the results. However, the results of this study are applicable to non‐Japanese PCOS with phenotype A and phenotype D interpreted by the Rotterdam criteria.

The incidence of uterine anomalies is suspected to be 3%–5% among infertile women, and a septate uterus is known to have higher risks of early spontaneous abortions or obstetrical complications.^20^, ^21^ Even an arcuate uterus is associated with an increased risk of preterm birth and fetal growth restriction.^22^ Several studies have shown elevated incidences of uterine anomalies in women with PCOS. Ugur et al.^7^ in a retrospective study reported that septate uteri were more prevalent in such women. Appelman et al.^8^ found a significant relationship between PCOS and Müllerian anomalies. Saleh and Shawky Moiety^9^ in a prospective study found that 31% of patients with PCOS had uterine anomalies, mainly an arcuate uterus, or, less frequently, a septate uterus, and 73% of patients with uterine anomalies were diagnosed with PCOS. Similarly, Ege et al.^10^ reported that septate and arcuate uteri had high prevalence in a PCOS group. Although several classification systems for Müllerian anomalies have been proposed, the American Fertility Society Classification published in 1988^23^ had mainly been utilized. To date, three diagnostic criteria have been proposed for the diagnosis of uterine anomalies: the European Society of Human Reproduction and Embryology/European Society for Gynaecological Endoscopy criteria^24^ have been reported to have a possibility of overestimation of the prevalence of septate uterus.^25^ The Congenital Uterine Malformation by Experts criteria‐2020^26^ and the ASRM classification‐2021^13^ might have improved diagnostic accuracy. A retrospective cohort study based on these newly developed criteria showed that patients with PCOS had fewer rates of normal uterine cavities and more frequent occurrences of septate or dysmorphic uterus, although the incidence of arcuate uterus was not increased.^11^ However, because these diagnostic criteria are not uniform in detail, discrepancies in diagnosis are inevitable, especially in distinguishing arcuate, septate, and normal uterine morphologies.

In this regard, 3D‐US has been established as a reliable and less invasive tool for the diagnosis of uterine anomalies.^27^ Recent advancements in image processing for 3D‐US have enabled us to evaluate correct uterine shapes with ease. Tracing a curved coronal plane allows accurate reconstruction of the uterine cavity. By rotating the reconstructed image, the interostial line becomes detectable in almost all cases. These features seem indispensable to perform this study. However, subjects with PCOS in this study had lesser septate uterus as compared to previous reports.^7^, ^8^, ^9^, ^10^, ^11^ We are not certain whether it was because of the limitation of this study, or Japanese characteristics, or the strict definition in the ASRM classification 2021. Moreover, this observational study did not intend to assess the relationship between uterine morphology and reproductive outcomes. We guess that poor reproductive outcomes of PCOS mainly depend on endocrine or metabolic features of the disease, not on the uterine morphology. However, the significance of this study was to reveal another common congenital feature of PCOS, even if it was just a small difference in uterine shape.

The etiology of uterine anomalies is not fully understood. The Müllerian ducts are formed in fetuses during Weeks 5 to 6 of gestation, along with the Wolffian duct, regardless of the sex of the fetus. In male embryos, Sertoli cells in testes produce AMH which regress the Müllerian ducts. On the contrary, in female embryos, AMH production from granulosa cells is not initiated until the late third trimester, the Müllerian ducts differentiate into fallopian tubes, uterus, and the upper portion of the vagina, under regulation by *Wnt* and *Hox* gene expression.^28^ The uterus is initially separated by a septum and then, fusion occurs. The outline of the uterus is complete by Weeks 12–13, and the septum regresses by week 20.^29^ Defects in the formation or regression of the Müllerian ducts can cause various uterine anomalies. Sex steroid hormones are one of epigenetic factors modulating *Hox* gene expression.^28^ Thus, it appears that prenatal androgen exposure in the first to early second trimester of pregnancy is possibly involved in the origin of uterine anomalies in women with PCOS.

## 5 CONCLUSIONS

These women with PCOS had deeper indentations of the uterine cavity and were more likely to develop an arcuate or septate uterus than were infertile women without PCOS. To our knowledge, this is the first study showing differences in uterine cavity shape in women with PCOS, independent from the diagnostic criteria or standard classifications of uterine anomalies. Limitations of this observational study are that the subjects comprise only women with infertility varying ages and that the evidence for prenatal androgen exposure is missing. However, it was estimated that women with PCOS had deeper indentation of the uterine cavity and were more likely to develop an arcuate or septate uterus. These findings might support evidence for the fetal origin of PCOS. Further prospective cohort studies are required to investigate the relationship between shapes of uterine cavity and other clues to assess prenatal androgen: the AGD or any information of mothers' menstrual or obstetrical history. Above all, further research to reveal the mechanism for the development of Müllerian anomalies in PCOS is essential in this field.

## FUNDING INFORMATION

There was no funding for the project.

## CONFLICT OF INTEREST STATEMENT

The authors declare no conflict of interest.

## HUMAN RIGHTS STATEMENTS AND INFORMED CONSENT

All procedures followed were in accordance with the ethical standards of the responsible committee on human experimentation and with the Helsinki Declaration of 1964 and its later amendments. Informed consent was obtained from all participants. The study protocol was approved by the Ethics Committee of our clinic. We certify that no persons other than the authors have made substantial contributions to the work.
